# Fall-related gait characteristics on the treadmill and in daily life

**DOI:** 10.1186/s12984-016-0118-9

**Published:** 2016-02-02

**Authors:** Sietse M. Rispens, Jaap H. Van Dieën, Kimberley S. Van Schooten, L. Eduardo Cofré Lizama, Andreas Daffertshofer, Peter J. Beek, Mirjam Pijnappels

**Affiliations:** Department of Human Movement Sciences, MOVE Research Institute Amsterdam, Vrije Universiteit Amsterdam, van der Boechorststraat 9, Amsterdam, 1081 BT The Netherlands; Department of Medicine (Royal Melbourne Hospital), The University of Melbourne, Melbourne, Australia

**Keywords:** Gait, Accidental falls, Elderly, Activities of daily living, Physical fitness, Accelerometry

## Abstract

**Background:**

Body-worn sensors allow assessment of gait characteristics that are predictive of fall risk, both when measured during treadmill walking and in daily life. The present study aimed to assess differences as well as associations between fall-related gait characteristics measured on a treadmill and in daily life.

**Methods:**

In a cross-sectional study, trunk accelerations of 18 older adults (72.3 ± 4.5 years) were recorded during walking on a treadmill (Dynaport Hybrid sensor) and during daily life (Dynaport MoveMonitor). A comprehensive set of 32 fall-risk-related gait characteristics was estimated and compared between both settings.

**Results:**

For 25 gait characteristics, a systematic difference between treadmill and daily-life measurements was found. Gait was more variable, less symmetric, and less stable during daily life. Fourteen characteristics showed a significant correlation between treadmill and daily-life measurements, including stride time and regularity (0.48 < *r* < 0.73; *p* < 0.022). No correlation between treadmill and daily-life measurements was found for stride-time variability, acceleration range and sample entropy in vertical and mediolateral direction, gait symmetry in vertical direction, and stability estimated as the local divergence exponent by Rosenstein’s method in mediolateral direction (*r* < 0.16; *p >* 0.25).

**Conclusions:**

Gait characteristics revealed less stable, less symmetric, and more variable gait during daily life than on a treadmill, yet about half of the characteristics were significantly correlated between conditions. These results suggest that daily-life gait analysis is sensitive to static personal factors (i.e., physical and cognitive capacity) as well as dynamic situational factors (i.e., behavior and environment), which may both represent determinants of fall risk.

**Electronic supplementary material:**

The online version of this article (doi:10.1186/s12984-016-0118-9) contains supplementary material, which is available to authorized users.

## Background

Quality of gait, as characterized among others by the stability, symmetry and variability of its kinematics, has been found to be associated with fall risk [1–6]. In this context, gait quality is often assessed in the laboratory, under standardized conditions so as to minimize any variance that does not reflect the individual’s walking ability, with treadmill walking at a fixed gait speed as the most controlled form. At the other extreme, gait quality is assessed in daily life, where outcomes may not only be influenced by the more static personal characteristics, but also by behavioral and environmental factors that vary dynamically from situation to situation. So in contrast to treadmill walking, people are free to adapt their speed at will and to divert their attention, but may also need to negotiate any obstacles and adjust their gait for turns during over ground walking in daily life. In other words, laboratory assessment may for example reflect how stable someone can walk at a given speed under near-optimal conditions, whereas daily-life assessment might reflect how fast someone chooses to walk under the experienced environmental conditions. Viewed in this light, it is surprising that assessments in the laboratory [1–3] as well as in daily-life [3–6] result in gait characteristics that are predictive of fall risk. However, not for all characteristics do the findings of their relation with fall risk agree between the laboratory and daily life. For example, in mediolateral (ML) direction, stride regularity, root-mean-square (RMS) acceleration and gait symmetry were found to discriminate between fallers and non-fallers in the laboratory, but not in daily life [7, 8].

For the use of gait quality characteristics in fall risk prediction models, it can be questioned whether and how these characteristics differ or agree between measurements in the laboratory or in daily life. While some of the gait characteristics used in fall risk prediction were reported to be correlated between settings, to our knowledge no comprehensive comparison between the two has been made to date. Such a comparison could also facilitate the interpretation of differences and agreement found between predictive values of gait characteristics obtained in different settings with respect to fall risk. We therefore compared a comprehensive set of fall-related gait characteristics determined during treadmill and daily-life gait. We selected characteristics that had previously been shown to be associated with fall risk. Since we expected individual walking ability to influence outcomes in both cases, we hypothesized measures obtained in the two settings to contain common information reflected by positive correlations between settings. Furthermore, situational effects were expected to influence gait characteristics in daily life, and we hypothesized this to cause systematic differences, with gait in daily life being less stable, less symmetric and more variable.

## Methods

### Participants

This study was part of a larger cohort study on Fall Risk Assessment in Older Adults (FARAO), focusing on the predictive value of gait characteristics derived from trunk accelerations in daily life for fall risk [6, 8]. A subgroup of 18 older adults (72.3 ± 4.5 years, 11 males and 7 females, 1.73 ± 0.09 m; mean ± standard deviation) participated in an additional study in the laboratory [9]. Inclusion criteria for the FARAO study were an age between 65 and 99 years, being able to walk 20 m, with a walking aid if needed and having a mini mental state examination score (MMSE) of 19 or higher. For the laboratory study these criteria were extended with having an MMSE of 25 or higher and no self-reported musculoskeletal or neurological conditions or use of medication that could have affected balance. All participants gave informed consent for both studies. The medical ethical committee of the VU University Medical Center Amsterdam (#2010/290, daily-life study) and the ethical committee of the Faculty of Human Movement Sciences, VU University Amsterdam (#2011/48, laboratory study), approved the study protocol.

### Protocol

Daily-life accelerations were collected with a portable tri-axial accelerometer (DynaPort MoveMonitor, McRoberts, The Hague, The Netherlands), which was attached over the lumbar spine with an elastic belt around the waist. The accelerometer’s range was set from –6 g to 6 g while its sample rate was set to 100 samples/s. Participants were invited to wear the accelerometer for one whole week at all times, except during showering and other aquatic activities to avoid damage to the instrument.

Trunk accelerations during treadmill walking were collected with a tri-axial inertial sensor, measuring accelerations and angular velocity (DynaPort Hybrid, McRoberts, The Hague, The Netherlands), of which only the accelerations were used in this study. The sensor’s range and sample rate were equal to those in the daily-life measurements. The inertial sensor was attached in the same position as during daily life measurements, i.e., over the lumbar spine with an elastic belt around the waist. Participants walked on the treadmill for 5 min at 1.2 m/s. As a safety precaution, participants wore a harness around their upper trunk, attached to the ceiling with a cable that provided enough slack not to interfere with walking.

### Data analysis

A comprehensive set of gait characteristics was estimated from the daily-life data and from the treadmill data: stride time by autocorrelation , stride time variability by time intervals between peaks in the estimated vertical position [5, 10], stride regularity as the autocorrelation [11], RMS acceleration by standard deviation [12], acceleration range as the difference between the minimum and maximum accelerations [3], gait symmetry as the harmonic ratio [12], local dynamic stability (Local Divergence Exponent) [5, 10] by the methods of Wolf [13] and Rosenstein [14], low frequency percentage [5, 10] with thresholds of 0.7, 10 and 0.7 Hz for VT, ML and AP directions, respectively, gait smoothness as the index of harmonicity [15], dominant frequency’s amplitude [3] as another measure for regularity, and sample entropy [16]. General descriptions of these characteristics have been presented in our previous studies [5, 10], except for sample entropy, which was determined by using 5 consecutive data points and 0.3 as the radius of tolerance [8]. All selected gait characteristics have previously been shown to be associated with past or future fall incidence or with clinical measures of fall risk in previous studies [3, 5–8, 17].

For the treadmill data, the first and last minute were discarded to avoid any transients, leaving three minutes of steady-state gait data for analysis. For the daily-life data, we first identified the locomotion episodes that lasted at least 10 s, using a validated algorithm [18] developed by the manufacturer of the sensors (McRoberts, The Hague, The Netherlands). For all data, gait characteristics were estimated from multiple 10-s epochs over which the median was taken. For treadmill walking, 3 min resulted in 18 epochs of 10 s. From daily-life gait episodes, we selected as many non-overlapping epochs of 10 s as possible, with any unused time equally divided over the beginning and end of the episode.

In addition to the characteristics listed above, we estimated gait speed [19], both during treadmill walking and daily life. Since speed was fixed during treadmill walking and since gait speed may affect gait characteristics, we added a comparison of treadmill characteristics with speed-matched epochs in daily life. Daily-life epochs were selected if their estimated speed was between 90 % and 110 % of the estimated treadmill speed. The range was scaled to the treadmill speed estimated with the same algorithm rather than the actual fixed treadmill speed, since we expected that a large part of the speed-estimate errors would be determined by individual differences in gait patterns.

### Statistics

First, deviations from normality of the gait-characteristic estimates were tested by the Kolomogorov-Smirnov test. Subsequently, we tested for systematic differences between treadmill and daily-life estimates. To this end, we applied a Wilcoxon’s signed rank test since equality of variance between settings was frequently violated. Pearson correlations were used to assess to what extent treadmill and daily-life measurements provided common information, except for the low-frequency percentage in VT direction, which was not normally distributed according to the Kolomogorov-Smirnov test, and for which Spearman correlation was used. Significance of positive correlation coefficients was tested for the hypothesis that they were greater than zero, rather than different from zero; this implies that *p*-values for positive correlations are adjusted by halving them and they range from 0 to 0.5, whereas *p*-values for negative correlations would range from 0.5 to 1. For each type of comparison, the significance level of 0.05 was adjusted using the Hochberg-Benjamini correction for multiple comparisons [20], which is appropriate when testing a set of related hypotheses.

## Results

**Fig. 1:**
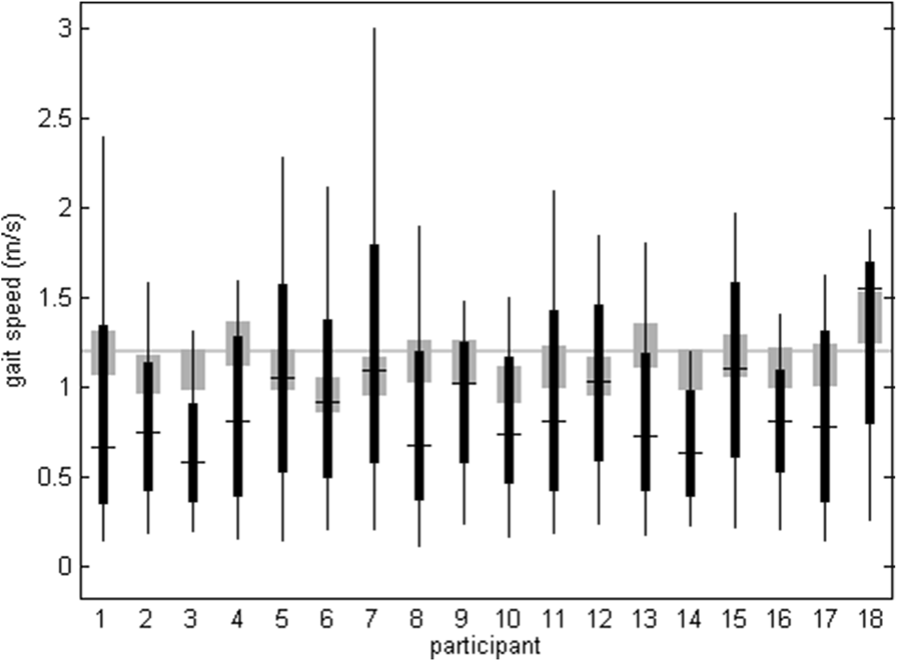
Box plot of gait speed estimates for 10-s epochs in daily life. For all individual participants, the ranges from minimum to maximum (vertical lines), the 25th to 75th percentiles (black rectangles) and the median (black horizontal lines) are shown. The grey rectangles indicate 90 % to 110 % of the treadmill speed estimated by the estimator used for daily life data, which was the range used for the speed-matched analysis. For reference, the treadmill speed setting (1.2 m/s) is plotted as a horizontal grey line

The daily-life recordings contained, on average, 1755 (range 683 – 3328 ) epochs of 10 s of locomotion per participant, which decreased to 330 (range 74 – 814) when selecting only speed-matched epochs [5, 10]. Details about the estimated gait speeds on the treadmill and in daily life are provided in Fig. 1.

Twenty-five of the 32 gait characteristics displayed a systematic difference between treadmill and daily-life estimates (Table 1). Gait in daily life was characterized by larger variability (stride time variability and low frequency percentage < 0.7 Hz), less regularity (stride regularity and the dominant frequency’s amplitude), less symmetry (harmonic ratio) and lower local dynamic stability (higher Local Divergence Exponent). No significant systematic difference between settings was found for estimates of RMS acceleration in vertical (VT) and anteroposterior (AP) direction, acceleration range in VT and ML direction, low frequency percentage below 10 Hz in ML direction, the index of harmonicity in AP direction, and sample entropy in VT direction.

**Table 1:** Mean and standard deviations (SD) of estimates based on treadmill, overall daily-life, and speed-matched daily-life gait, Wilcoxon’s signed rank test *p*-values and correlation coefficient *r* (*p*-value) between treadmill and daily-life settings

Gait characteristic	Treadmill mean (SD)	Daily-life mean (SD)	Speed-matched daily-life mean (SD)	Systematic difference *p*-value	Syst. diff. *p*-value (speed-matched)	correlation *r* (*p*)	correlation *r* (*p*) (speed-matched)
Stride Time (s)	1.09 (0.07)	1.15 (0.11)	1.12 (0.09)	**0.009**	**0.019**	**0.58 (0.006)**	**0.80 (<0.001)**
Stride Time Variability (s)	0.02 (0.00)	0.07 (0.04)	0.03 (0.01)	**<0.001**	**<0.001**	0.09 (0.362)	–0.08 (0.624)
Stride Regularity VT	0.85 (0.07)	0.55 (0.18)	0.68 (0.12)	**<0.001**	**<0.001**	**0.62 (0.003)**	0.26 (0.153)
Stride Regularity ML	0.69 (0.09)	0.40 (0.14)	0.42 (0.12)	**<0.001**	**<0.001**	**0.56 (0.008)**	0.44 (0.033)
Stride Regularity AP	0.78 (0.07)	0.48 (0.16)	0.55 (0.15)	**<0.001**	**<0.001**	**0.48 (0.021)**	0.27 (0.143)
RMS Acceleration VT (ms^-2^)	2.32 (0.41)	2.24 (0.82)	2.42 (0.43)	0.640	0.140	0.45 (0.032)	**0.80 (<0.001)**
RMS Acceleration ML (ms^-2^)	1.49 (0.26)	1.31 (0.27)	1.34 (0.21)	**0.027**	**0.030**	0.26 (0.146)	0.38 (0.062)
RMS Acceleration AP (ms^-2^)	1.45 (0.28)	1.46 (0.36)	1.57 (0.25)	0.829	**<0.001**	**0.73 (<0.001)**	**0.88 (<0.001)**
Acceleration Range VT (ms^-2^)	11.75 (2.68)	13.92 (4.00)	13.78 (2.34)	0.054	**0.010**	0.16 (0.264)	0.31 (0.107)
Acceleration Range ML (ms^-2^)	10.25 (2.04)	9.72 (2.39)	10.70 (2.18)	0.498	0.510	–0.06 (0.600)	0.08 (0.381)
Acceleration Range AP (ms^-2^)	8.56 (1.97)	10.38 (2.94)	10.94 (1.98)	**0.004**	**<0.001**	**0.61 (0.004)**	**0.71 (<0.001)**
Gait symmetry (Harmonic Ratio) VT	2.80 (0.56)	1.71 (0.39)	2.14 (0.35)	**<0.001**	**<0.001**	0.14 (0.283)	0.20 (0.212)
Gait symmetry (Harmonic Ratio) ML	2.01 (0.41)	1.46 (0.19)	1.57 (0.23)	**<0.001**	**<0.001**	**0.50 (0.016)**	**0.50 (0.018)**
Gait symmetry (Harmonic Ratio) AP	2.53 (0.48)	1.48 (0.32)	1.79 (0.38)	**<0.001**	**<0.001**	0.32 (0.100)	0.11 (0.335)
Local Divergence Exponent Wolf VT (s^-1^)	0.83 (0.25)	1.42 (0.32)	1.24 (0.27)	**<0.001**	**<0.001**	**0.59 (0.005)**	0.46 (0.026)
Local Divergence Exponent Wolf ML (s^-1^)	1.31 (0.31)	1.73 (0.23)	1.74 (0.19)	**<0.001**	**<0.001**	**0.51 (0.015)**	**0.52 (0.013)**
Local Divergence Exponent Wolf AP (s^-1^)	1.02 (0.23)	1.62 (0.26)	1.51 (0.29)	**<0.001**	**<0.001**	**0.55 (0.009)**	0.44 (0.035)
Local Divergence Exponent Rosenstein VT (s^-1^)	0.61 (0.10)	0.76 (0.09)	0.77 (0.10)	**<0.001**	**<0.001**	0.28 (0.132)	0.31 (0.108)
Local Divergence Exponent Rosenstein ML (s^-1^)	0.57 (0.08)	0.64 (0.07)	0.62 (0.07)	**0.008**	0.056	0.01 (0.490)	–0.00 (0.504)
Local Divergence Exponent Rosenstein AP (s^-1^)	0.54 (0.09)	0.65 (0.06)	0.62 (0.06)	**<0.001**	**<0.001**	0.21 (0.201)	0.37 (0.068)
Low frequency percentage VT < 0.7 Hz (%)	0.08 (0.09)	0.22 (0.15)	0.16 (0.11)	**<0.001**	**<0.001**	–0.15 (0.722)	–0.15 (0.722)
Low frequency percentage ML < 10 Hz (%)	85.18 (5.96)	86.70 (5.11)	83.67 (6.12)	0.308	0.292	0.39 (0.052)	**0.52 (0.013)**
Low frequency percentage AP < 0.7 Hz (%)	0.82 (0.38)	4.20 (2.99)	2.63 (1.56)	**<0.001**	**<0.001**	**0.51 (0.015)**	0.12 (0.313)
Gait Smoothness (Index of Harmonicity) VT	0.67 (0.10)	0.59 (0.11)	0.70 (0.09)	**0.007**	0.188	**0.53 (0.013)**	**0.58 (0.005)**
Gait Smoothness (Index of Harmonicity) ML	0.10 (0.09)	0.26 (0.17)	0.22 (0.13)	**<0.001**	**<0.001**	0.37 (0.068)	**0.52 (0.014)**
Gait Smoothness (Index of Harmonicity) AP	0.57 (0.10)	0.55 (0.10)	0.59 (0.07)	0.486	0.286	0.39 (0.057)	**0.56 (0.008)**
Dominant Frequency’s Amplitude VT	0.82 (0.14)	0.66 (0.14)	0.78 (0.13)	**<0.001**	0.208	**0.65 (0.002)**	**0.49 (0.019)**
Dominant Frequency’s Amplitude ML	0.45 (0.12)	0.38 (0.09)	0.31 (0.07)	**0.036**	**<0.001**	0.19 (0.220)	0.44 (0.034)
Dominant Frequency’s Amplitude AP	0.63 (0.13)	0.49 (0.10)	0.53 (0.12)	**0.002**	**0.015**	0.20 (0.210)	0.24 (0.173)
Sample Entropy VT	0.26 (0.05)	0.24 (0.03)	0.23 (0.04)	0.092	**0.029**	–0.09 (0.631)	0.24 (0.172)
Sample Entropy ML	0.40 (0.07)	0.35 (0.04)	0.37 (0.05)	**0.025**	0.108	–0.00 (0.505)	0.07 (0.392)
Sample Entropy AP	0.29 (0.08)	0.25 (0.05)	0.24 (0.05)	**0.027**	**0.003**	**0.61 (0.003)**	**0.66 (0.001)**

Significant values are printed in bold face (*p* < 0.039 and *p* < 0.038 for systematic differences, and *p* < 0.022 and *p* < 0.019 for correlation coefficients, for overall and speed-matched daily-life gait, respectively)

**Fig. 2:**
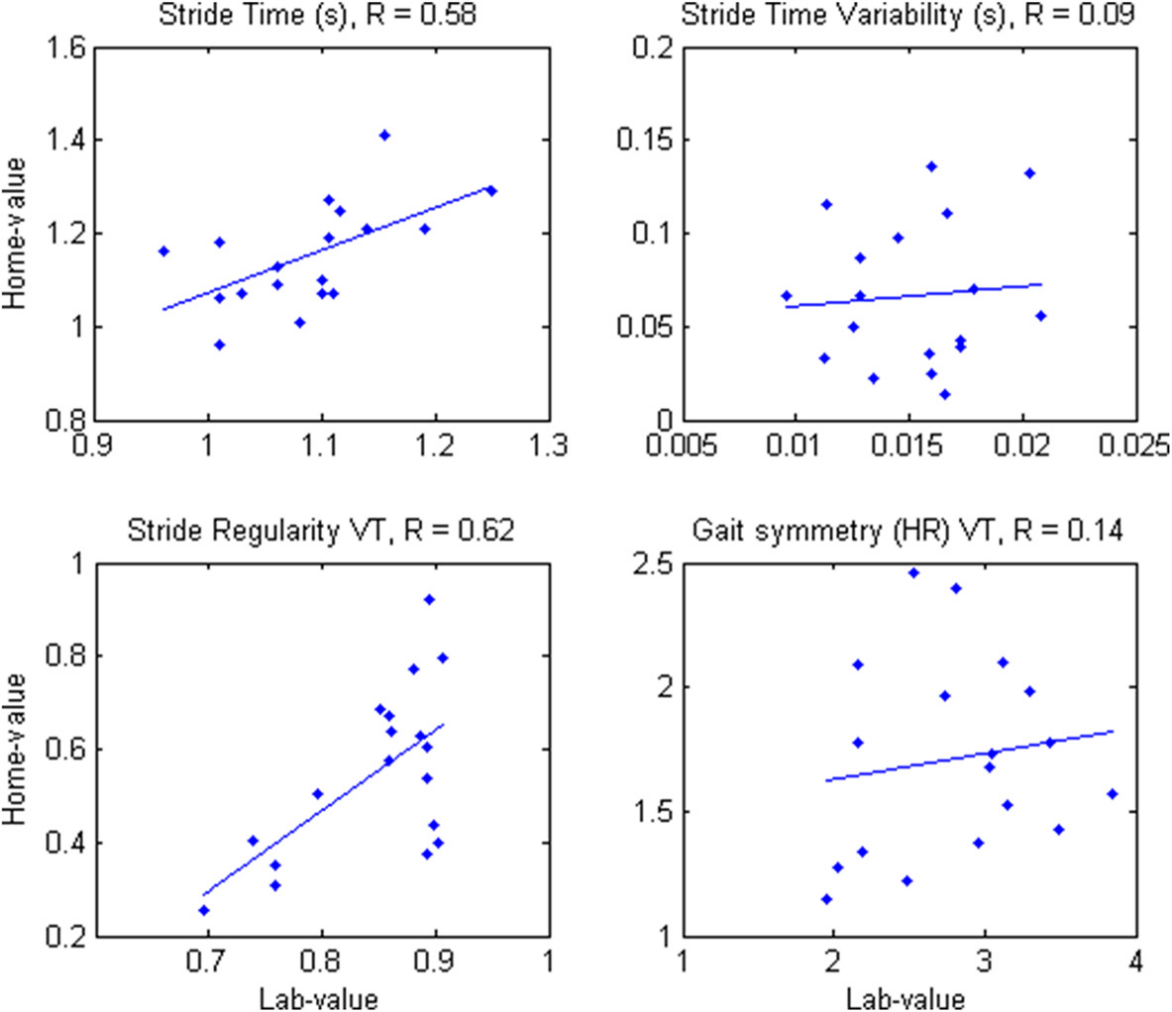
Scatter plots (blue dots) for estimated characteristics on the treadmill (x-axis) versus daily life (y-axis). A linear fit is plotted as a blue line

For 14 of the characteristics we found a significant positive correlation between treadmill and daily-life estimates (Table 1). However, we did not find a significant correlation between treadmill and daily-life estimates for stride time variability, RMS acceleration and acceleration range in VT and ML directions, gait symmetry in VT and AP directions, local dynamic stability in terms of the Local Divergence Exponent as estimated by Rosenstein’s method, low frequency percentage under 10 Hz in ML direction, gait smoothness in ML and AP direction, the dominant frequency’s amplitude in ML and AP directions, and sample entropy in VT and ML directions. Scatter plots of the treadmill versus daily-life estimates can be found in Fig. 2 and in the Additional file 1 and Additional file 2.

The speed-matched comparison revealed similar patterns of systematic differences and correlations between treadmill and daily-life gait characteristics as the overall comparison. However, some characteristics showed clear changes in the strengths of correlations or the size of systematic differences. Stride time, RMS acceleration and acceleration range, low-frequency percentage in VT and ML direction, and gait smoothness in each direction had a stronger correlation, whereas stride regularity in each direction, the Local Divergence Exponents in VT and AP directions and the low-frequency percentage in AP direction had a lower correlation after selecting the speed-matched epochs in daily life. Systematic differences were typically smaller for the speed-matched comparison than for the comparison with all daily life epochs, with the exception of the RMS acceleration and acceleration range in AP direction and the dominant frequency’s amplitude in ML directions, which had substantially larger systematic differences.

## Discussion

We compared gait characteristics estimated from measurements in daily life with those obtained under standardized laboratory conditions, i.e., during treadmill walking at a fixed speed. We found systematic differences between the two settings for most of the gait characteristics and found significant correlations between settings for about half of the gait characteristics.

The systematic differences between settings may reflect an interaction of individual and environmental factors on the selection of instantaneous gait patterns, in terms of for example adapting speed, varying heading, or stepping over obstacles, which were controlled on the treadmill but not in daily life. An important factor influencing these findings might be the difference in gait speed between treadmill and daily-life settings. On the treadmill, gait speed was fixed at 1.2 m/s, close to the average preferred speed of a large cohort studied by Studenski and co-workers [21], whereas it was on average lower and varied widely within and between our subjects in the daily life measurements (as can be seen in Fig. 1). Gait speed is known to have substantial effects on many gait parameters studied here (e.g.,[11, 22, 23]). We investigated this effect by comparing treadmill gait and daily-life gait with matched estimated speed. The systematic difference of treadmill gait with speed-matched daily-life gait was typically smaller than with all daily-life gait, which is in line with the assumption that part of the systematic difference was caused by speed differences. However, RMS and range or the accelerations showed larger systematic differences in the speed-matched comparison, which is not as expected considering that these measures are strongly dependent on gait speed [12].

We incorporated gait characteristics that had previously been shown to be associated with fall risk. It is, therefore, reasonable to assume that these reflect physical and cognitive capacities of the individual to generate a stable gait pattern, overcome perturbations and prevent falls. Many of the gait characteristics did show a significant correlation between laboratory and daily-life settings. We propose that the common information between the two settings is determined by personal factors (i.e., the individual’s physical and cognitive capacity). This suggests that the characteristics that did not correlate significantly between settings are more strongly influenced by situational factors. These parameters will be discussed below.

Stride time variability was not significantly correlated between settings. Stride time variability was solely found to be associated with fall risk when estimated under controlled conditions [2, 24], and not when estimated in daily life [5, 6]. The association found under controlled conditions indicates that this parameter reflects an important aspect of the individual’s capacity. Apparently this information is obscured in daily-life measurements due to the large effects of situational factors, as suggested previously [25].

Parameters indicating gait intensity, such as the RMS and range of the accelerations showed inconsistent correlations. RMS and range of ML and VT accelerations were not significantly correlated between settings, while for AP acceleration they were. The ML direction may predominantly reflect turning [6], which may have had a major influence on the ML intensity parameters in daily life, whereas turns were obviously absent on the treadmill. Amplitude of movement in the VT direction is highly related to gait speed [19], which was equal for subjects on the treadmill but not in daily life and which could therefore explain the absence of a correlation. This explanation is supported by the speed-matched analysis, in which we found a stronger correlation between settings for the RMS acceleration in VT direction (Table 1). The same dependency on gait speed holds for acceleration in the AP direction, but differences in sensitivity to spatiotemporal parameters such as step time and step length variability [26] might cause the differences between VT and AP directions of correlations found between treadmill and daily-life.

Another parameter showing different results for different directions was gait symmetry estimated by the harmonic ratio; correlations were non-significant in VT and AP directions, while gait symmetry in the ML direction was significantly correlated between settings. The non-significant correlation for gait symmetry in VT direction is surprising because previous studies, both in daily life and in the laboratory, revealed its association with fall risk [6, 8, 27] . These latter findings were obtained during over-ground instead of treadmill walking. Since others did not find an association between fall risk and gait symmetry as determined from treadmill walking [1], this may indicate that treadmill walking strongly affects gait symmetry in VT and AP directions, rendering it less representative for personal fall risk factors.

Local dynamic stability parameters as estimated by Rosenstein’s method [14] did not significantly correlate between treadmill and daily life, while those estimated by Wolf’s method [13] did. Although both methods have been designed to estimate the same concept, they apparently yield different estimates. Possibly the two estimators were affected differentially by other aspects of the measured signals than local dynamic stability. Further research is needed to address this issue.

The low-frequency percentage below 10 Hz in the ML direction has been suggested to reflect the ability to quickly respond to balance disturbances [5], which may be called upon more frequently during daily life than on a treadmill. The different requirements between walking on a treadmill and in daily life might thus explain the lack of correlation between settings.

Gait smoothness in ML and AP directions was not significantly correlated between treadmill and daily life settings. After selecting only the speed-matched epochs in daily life, these parameters did show a significant correlation, indicating their gait-speed dependency.

The dominant frequency’s amplitude in ML direction, a measure of gait consistency, was not significantly correlated between laboratory and daily-life settings. As we found a significant correlation for stride regularity, which is estimated from the signal’s auto-correlation and is similar to consistency, this raises the question how these two characteristics are different. An important difference is that stride regularity is based on stride time, whereas the dominant frequency in ML direction is not always the stride frequency but can also be an odd higher harmonic thereof. A closer look at the dominant frequencies in ML direction showed that on the treadmill they were on average typically between 4.2 and 5.1 Hz (14 of 18 participants), while in daily life they were typically below 3 Hz (12 of 18 participants). These differences in dominant frequencies may have had a significant impact on its amplitude, and may explain why we found no significant correlation between settings for this parameter.

The last characteristic that did not show a significant correlation between treadmill and daily-life walking was sample entropy in VT and ML directions. For these directions, the studies on fall risk do not disagree on predictive value of treadmill versus daily-life gait [1, 8]. However, sample entropy in AP direction was correlated between the two settings, while for this parameter treadmill-gait estimates did discriminate fallers and non-fallers [1], while daily-life gait estimates did not [8].

Although gait characteristics are usually strongly dependent on speed, correlations between gait characteristics on the treadmill and in daily life were, contrary to expectations, not consistently higher after selecting the epochs in daily life that matched the treadmill in estimated speed (Table 1). While the RMS and range of the accelerations revealed stronger correlations between settings after matching the estimated speeds, other characteristics’ associations with the gait-speed estimator used were possibly not sufficient to let the speed matching consistently strengthen the correlations between treadmill and daily-life gait.

Although we were able to systematically compare gait characteristics obtained in a controlled setting with those obtained in daily life, this study does have some limitations. First, the estimation of gait characteristics from daily-life measurements may have caused substantial noise in our estimates. Gait characteristics in daily life were more variable over time than gait characteristics in the laboratory. To compensate for this, considerably more data were obtained in daily life and only the median estimate was retained. We therefore do not expect this to have had a major influence on our results, given the reliability of the daily-life estimates shown previously [5]. Furthermore, we used treadmill walking as the most controlled commonly used setting for gait assessment. One may expect that other conditions such as over-ground walking or treadmill walking at preferred walking speed, which are less strictly controlled, would show better agreement with daily-life gait at least for some of the characteristics. For example, Dingwell et al. [28] showed that gait variability and stability differ between over-ground and treadmill walking. As we pointed out in the discussion of symmetry measures, some of the measures might have been significantly correlated if we had used over-ground walking as our controlled condition. The effects of a specific constraint such as walking speed and the differences between over-ground walking in the laboratory and in daily life might be further uncovered if future research would include a condition of controlled over-ground walking and or treadmill walking at preferred or multiple walking speeds.

Finally we would like to point out that although our results suggest that daily-life gait analysis provides information on walking ability as well as behavioral and environmental influences on gait, and although all of these are likely to have a bearing on fall risk, it is not necessarily superior over gait assessment in the laboratory in prediction of fall risk. The results do indicate that a direct comparison of the predictive ability of parameters obtained in the laboratory and in daily life is warranted. In addition, the participants in this study, being able to walk on the treadmill at 1.2 m/s, were relatively fit older adults, which may limit their representativeness for fall-risk related interpretations. For assessment of the potential of the gait characteristics from either or both settings in fall risk prediction, further research on large cohorts is necessary.

## Conclusion

Estimates of fall-related gait characteristics from trunk accelerations typically displayed a systematic difference between measurements on a treadmill and in daily life. Half of the characteristics analyzed showed significant but at best moderate correlations between settings, whereas others were not significantly correlated, even when matching for comparable gait speed episodes from daily life. Our results indicate that daily-life gait is sensitive to personal factors (i.e., physical and cognitive capacity) as well as situational factors (i.e., behavior and environment), which may both represent determinants of fall risk.

## Additional files

Additional file 1:
**Scatter plots (blue dots) for estimated characteristics on the treadmill (x-axis) versus daily life (y-10.1186/s12984-016-0118-9 axis).** A linear fit is plotted as a blue line. (PNG 23 kb) Additional file 2
**Scatter plots (blue dots) for estimated characteristics on the treadmill (x-axis) versus daily life (y-10.1186/s12984-016-0118-9 axis).** A linear fit is plotted as a blue line. (PNG 23 kb)

## References

[1] Riva F, Toebes MJ, Pijnappels M, Stagni R, van Dieën JH (2013). Estimating fall risk with inertial sensors using gait stability measures that do not require step detection. Gait Posture.

[2] Toebes MJP, Hoozemans MJM, Furrer R, Dekker J, van Dieën JH (2012). Local dynamic stability and variability of gait are associated with fall history in elderly subjects. Gait Posture.

[3] Weiss A, Brozgol M, Dorfman M, Herman T, Shema S, Giladi N (2013). Does the evaluation of gait quality during daily life provide insight into fall risk? A novel approach using 3-day accelerometer recordings. Neurorehabil Neural Repair.

[4] Marschollek M, Rehwald A, Wolf K, Gietzelt M, Nemitz G, Meyer Zu Schwabedissen H (2011). Sensor-based fall risk assessment - an expert ‘to go’. Method Inform Med.

[5] Rispens SM, van Schooten KS, Pijnappels M, Daffertshofer A, Beek PJ, van Dieën JH (2015). Identification of fall risk predictors in daily life measurements: gait characteristics’ reliability and association with self-reported fall history. Neurorehabil Neural Repair.

[6] van Schooten KS, Pijnappels M, Rispens SM, Elders PJM, Lips P, van Dieën JH. Ambulatory fall-risk assessment: Amount and quality of daily-life gait predict falls in older adults. J Gerontol A Biol Sci Med Sci. 2015. doi:10.1093/gerona/glu225 10.1093/gerona/glu225 25568095

[7] Howcroft J, Kofman J, Lemaire ED (2013). Review of fall risk assessment in geriatric populations using inertial sensors. J Neuroeng Rehabil.

[8] Rispens SM, van Schooten KS, Pijnappels M, Daffertshofer A, Beek PJ, van Dieën JH (2015). Do extreme values of daily-life gait characteristics provide more information about fall risk than median values?. JMIR research protocols.

[9] Lizama LEC, Pijnappels M, Rispens SM, Reeves NP, Verschueren SM, van Dieën JH (2015). Mediolateral balance and gait stability in older adults. Gait Posture.

[10] Rispens SM, Pijnappels M, van Schooten KS, Beek PJ, Daffertshofer A, van Dieën JH (2014). Consistency of gait characteristics as determined from acceleration data collected at different trunk locations. Gait Posture.

[11] Moe-Nilssen R, Helbostad JL (2004). Estimation of gait cycle characteristics by trunk accelerometry. J Biomech.

[12] Menz HB, Lord SR, Fitzpatrick RC (2003). Acceleration patterns of the head and pelvis when walking on level and irregular surfaces. Gait Posture.

[13] Wolf A, Swift JB, Swinney HL, Vastano JA (1985). Determining Lyapunov exponents from a time series. Phys D.

[14] Rosenstein MT, Collins JJ, De Luca CJ (1993). A practical method for calculating largest Lyapunov exponents from small data sets. Phys D.

[15] Lamoth CJC, Beek PJ, Meijer OG (2002). Pelvis–thorax coordination in the transverse plane during gait. Gait Posture.

[16] Richman JS, Moorman JR (2000). Physiological time-series analysis using approximate entropy and sample entropy. Am J Physiol-Heart C.

[17] Hamacher D, Singh NB, van Dieën JH, Heller MO, Taylor WR (2011). Kinematic measures for assessing gait stability in elderly individuals: a systematic review. J Royal Soc Interface.

[18] Dijkstra B, Kamsma Y, Zijlstra W (2010). Detection of gait and postures using a miniaturised triaxial accelerometer-based system: accuracy in community-dwelling older adults. Age Ageing.

[19] Zijlstra W, Hof AL (2003). Assessment of spatio-temporal gait parameters from trunk accelerations during human walking. Gait Posture.

[20] Benjamini Y, Hochberg Y (1995). Controlling the false discovery rate: a practical and powerful approach to multiple testing. J R Stat Soc.

[21] Studenski S, Perera S, Patel K, Rosano C, Faulkner K, Inzitari M (2011). Gait speed and survival in older adults. JAMA.

[22] Bruijn SM, van Dieën JH, Meijer OG, Beek PJ (2009). Is slow walking more stable?. J Biomech.

[23] Dingwell JB, Marin LC (2006). Kinematic variability and local dynamic stability of upper body motions when walking at different speeds. J Biomech.

[24] Hausdorff JM, Rios DA, Edelberg HK (2001). Gait variability and fall risk in community-living older adults: a 1-year prospective study. Arch Phys Med Rehabil.

[25] van Schooten KS, Rispens SM, Elders PJ, van Dieën JH, Pijnappels M (2014). Toward ambulatory balance assessment: Estimating variability and stability from short bouts of gait. Gait Posture.

[26] Moe-Nilssen R, Aaslund MK, Hodt-Billington C, Helbostad JL (2010). Gait variability measures may represent different constructs. Gait Posture.

[27] Doi T, Hirata S, Ono R, Tsutsumimoto K, Misu S, Ando H (2013). The harmonic ratio of trunk acceleration predicts falling among older people: results of a 1-year prospective study. J Neuroeng Rehabil.

[28] Dingwell JB, Cusumano JP, Cavanagh PR, Sternad D (2001). Local dynamic stability versus kinematic variability of continuous overground and treadmill walking. J Biomech Eng.

