# Supplementary figures and images for: Fall-related gait characteristics on the treadmill and in daily life
Source: J Neuroeng Rehabil. 2016 Feb 2;13:12. doi: 10.1186/s12984-016-0118-9 (PMC4736650; doi:10.1186/s12984-016-0118-9)

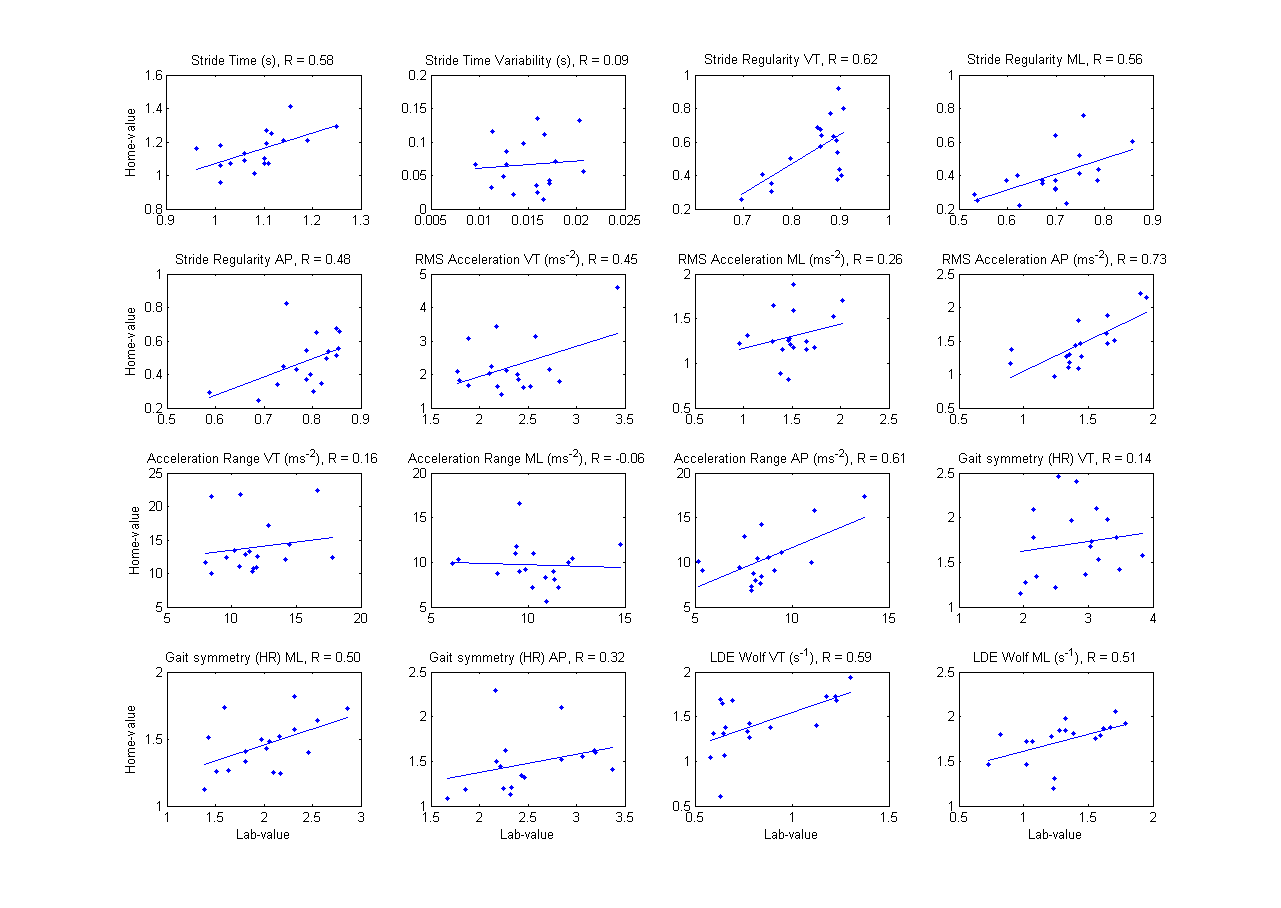

Supplement: Additional file 1: — Scatter plots (blue dots) for estimated characteristics on the treadmill (x-axis) versus daily life (y-10.1186/s12984-016-0118-9 axis). A linear fit is plotted as a blue line. (PNG 23 kb) [file 12984_2016_118_MOESM1_ESM.png]

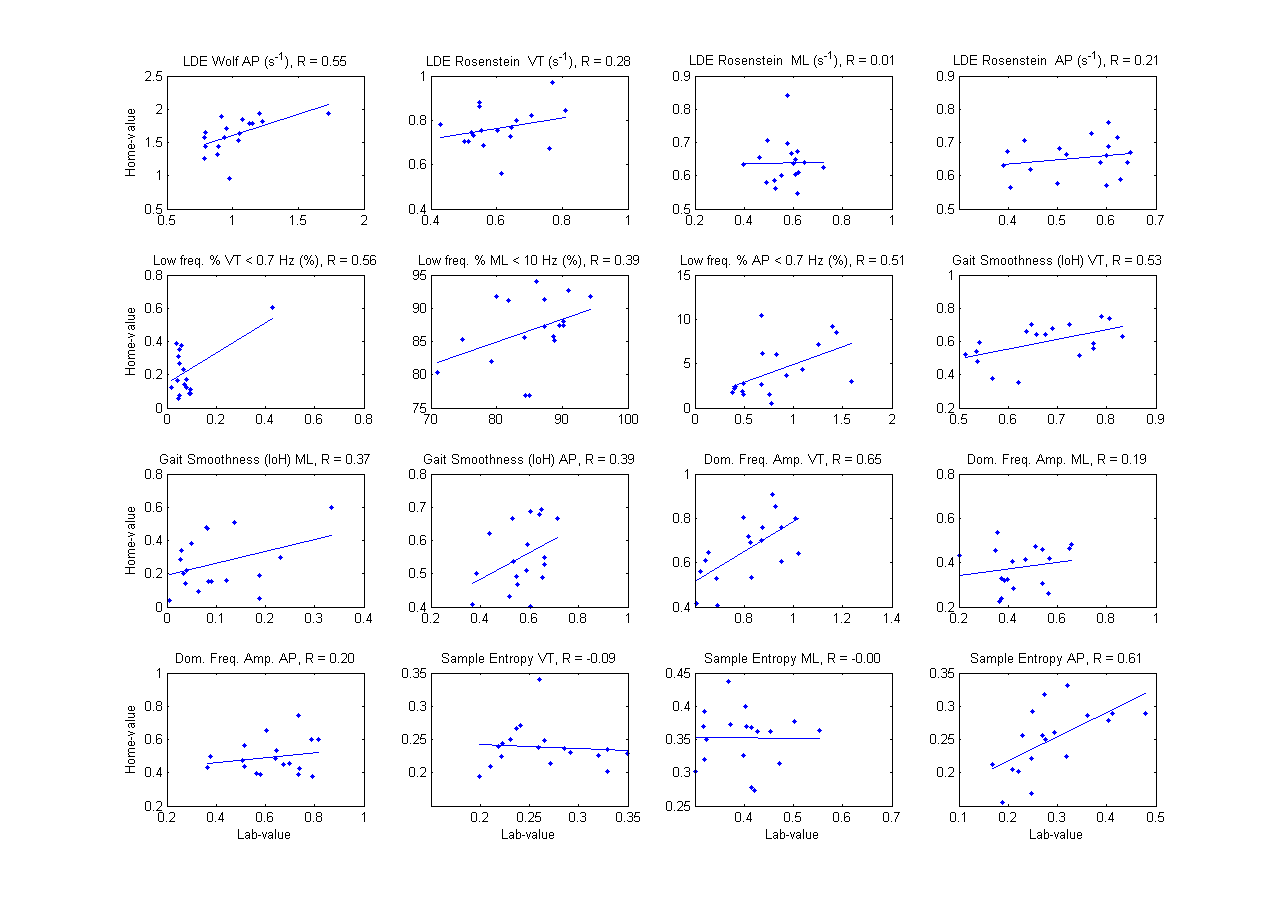

Supplement: Additional file 2 — Scatter plots (blue dots) for estimated characteristics on the treadmill (x-axis) versus daily life (y-10.1186/s12984-016-0118-9 axis). A linear fit is plotted as a blue line. (PNG 23 kb) [file 12984_2016_118_MOESM2_ESM.png]
